# Transcription Factor GFI1B in Health and Disease

**DOI:** 10.3389/fonc.2017.00054

**Published:** 2017-03-28

**Authors:** Eduardo Anguita, Francisco J. Candel, Alberto Chaparro, Juan J. Roldán-Etcheverry

**Affiliations:** ^1^Hematology Department, Hospital Clínico San Carlos, Instituto de Investigación Sanitaria San Carlos (IdISSC), Madrid, Spain; ^2^Department of Medicine, Universidad Complutense de Madrid (UCM), Madrid, Spain; ^3^Microbiology Department, Hospital Clínico San Carlos, Instituto de Investigación Sanitaria San Carlos (IdISSC), Madrid, Spain

**Keywords:** GFI1B, hematopoiesis, cancer, leukemia, platelets disease

## Abstract

Many human diseases arise through dysregulation of genes that control key cell fate pathways. Transcription factors (TFs) are major cell fate regulators frequently involved in cancer, particularly in leukemia. The *GFI1B* gene, coding a TF, was identified by sequence homology with the oncogene growth factor independence 1 (*GFI1*). Both GFI1 and GFI1B have six C-terminal C2H2 zinc fingers and an N-terminal SNAG (SNAIL/GFI1) transcriptional repression domain. Gfi1 is essential for neutrophil differentiation in mice. In humans, *GFI1* mutations are associated with severe congenital neutropenia. Gfi1 is also required for B and T lymphopoiesis. However, knockout mice have demonstrated that *Gfi1b* is required for development of both erythroid and megakaryocytic lineages. Consistent with this, human mutations of *GFI1B* produce bleeding disorders with low platelet count and abnormal function. Loss of *Gfi1b* in adult mice increases the absolute numbers of hematopoietic stem cells (HSCs) that are less quiescent than wild-type HSCs. In keeping with this key role in cell fate, *GFI1B* is emerging as a gene involved in cancer, which also includes solid tumors. In fact, abnormal activation of *GFI1B* and *GFI1* has been related to human medulloblastoma and is also likely to be relevant in blood malignancies. Several pieces of evidence supporting this statement will be detailed in this mini review.

Hematopoiesis, the process of formation of blood cellular components, provides an ideal model for cell differentiation of particular interest, as maturation block plays a crucial role in the pathogenesis of blood malignancies. It is regulated by transcription factors (TFs) that control gene expression, binding to specific DNA sequences and recruiting protein complexes that modify chromatin. Therefore, TFs are essential to establish a homeostatic balance, and they also play an essential role in disease.

Growth factor independence 1 (GFI1) and its homolog GFI1B are lineage-specific TFs required for hematopoiesis. *GFI1* was discovered as a proviral integration site that confers IL-2-independency in T-cells infected by Moloney murine leukemia virus and was mapped to chromosome 1 of the human genome (1p22) (1, 2) (Figure 1A). The *GFI1* paralog—*GFI1B*—was identified by its sequence homology with *GFI1* and mapped to chromosome 9 of the human genome (9q34.13) (3, 4).

**Figure 1:**
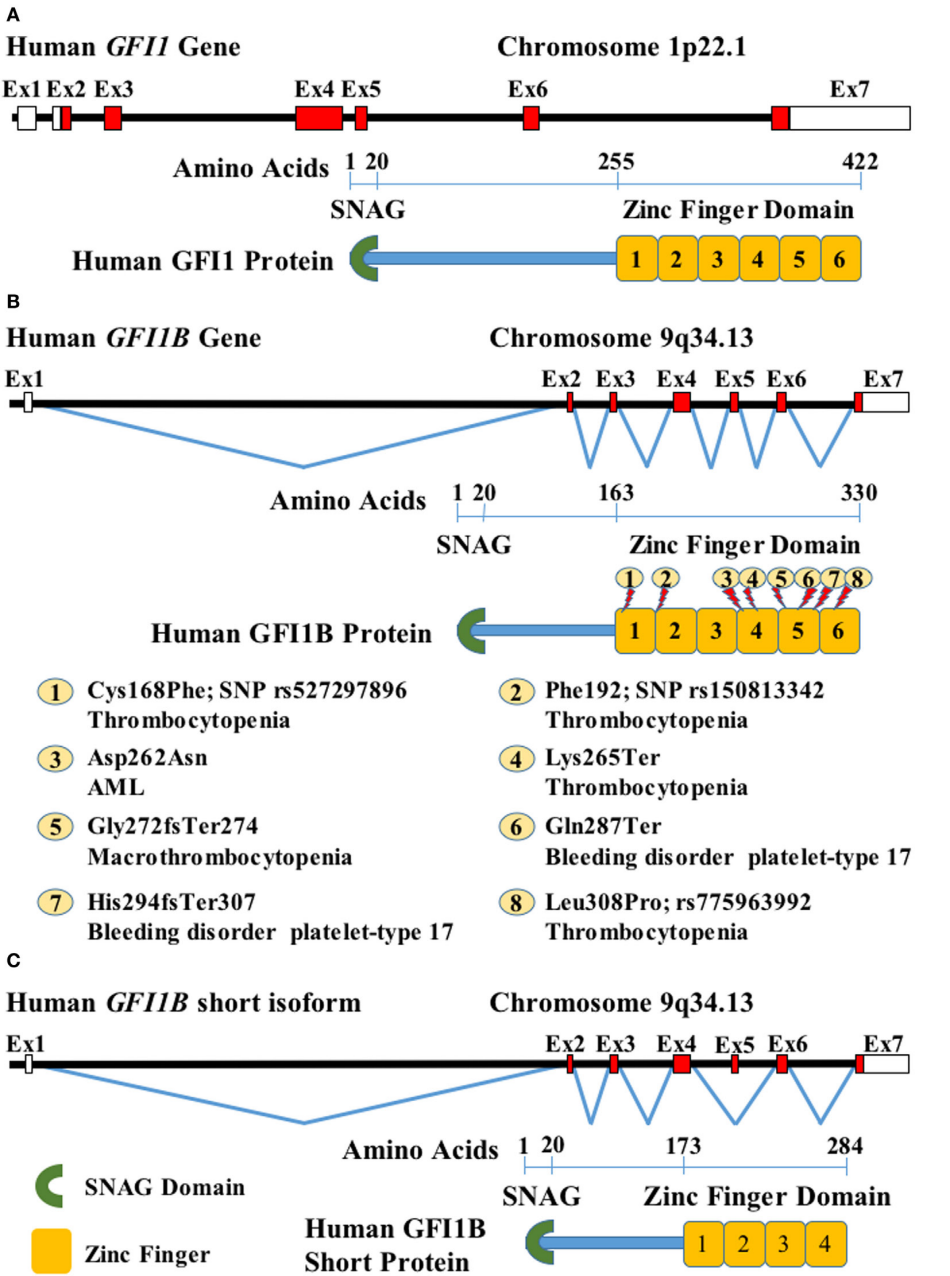
**Schematic representation of human growth factor independence 1 (*GFI1*) and *GFI1B* genes and corresponding protein domains: (A) diagram of human *GFI1* locus and protein**. On top, the black horizontal line represents the DNA sequence, red boxes on the sequence are coding *GFI1* exons (Ex), and white boxes indicate UTRs. Underneath, GFI1 protein domains are indicated (green crescent represents the SNAG domain, and orange boxes are zinc fingers), the numbers over the blue rule indicate the amino acid positions; **(B)** *GFI1B* locus and long protein isoforms are depicted as in panel **(A)**. The zigzag lines joining the Ex represent the splicing. Known mutations are shown on the protein and briefly described [acute myeloid leukemia (AML)]. **(C)** Short *GFI1B* splice variant and protein represented as in panels **(A,B)**.

## GFI1 and GFI1B Structure and Function

Growth factor independence 1 and GFI1B share two domains with over 95% identity. The conserved N-terminal SNAG domain contains 20 amino acids, which recruit proteins that modify histones (4–6). This domain has a nuclear localization motif and plays an important role in transcriptional repression through the binding of cofactors lysine-specific histone demethylase 1A (KDM1A, also known as LSD1) and RCOR1/2 (COREST) (6–8). However, the SNAG domain is not required for GFI1 interaction with corepressors euchromatic histone lysine methyltransferase 2 (EHMT2, G9A) and histone deacetylase HDAC 1 (9), suggesting that different portions of these proteins cooperate in gene repression, possibly with different peculiarities in each TF.

The C-terminal domain is formed by a highly conserved region with six C2H2 zinc fingers. Fingers 1, 2, and 6 are required for protein interaction, whereas fingers 3–5 are necessary to bind DNA at an AATC containing motif [TA**AATC**AC(T/A)GC(A/T)] (10, 11).

Between both domains, there is a less-characterized region that completely differs in both proteins, the function of which is still unknown.

This region is responsible for the size difference in both proteins: GFI1 has 422 amino acids (55 kDa), while GFI1B consists of 330 amino acids (37 kDa, CCDS6957) (Figure 1B). There is also a short 284 amino acid GFI1B isoform (CCDS48049) (Figure 1C) that lacks the first two zinc-finger domains as a result of an alternative splicing, skipping exon 5 (ENST00000372123.4).

Although GFI1 and GFI1B are similar in structure and share functional mechanisms, they show distinct cell expression patterns. Both GFI1 and GFI1B have an important role in the endothelial cell to hematopoiesis transition, the process by which endothelial cells become blood cells during the third wave of blood development. This generates the first hematopoietic stem cells (HSCs) in the intraembryonic aorta–gonad–mesonephros region, silencing the endothelial program. Interestingly, the expression pattern of both genes is different: *Gfi1* is specifically expressed within the dorsal aorta in endothelial cells and cells within emerging intra-aortic hematopoietic clusters, whereas *Gfi1b* expression is more associated with the fully formed intra-aortic hematopoietic clusters (12). This suggests that although both proteins can apparently compensate for the loss of one gene by the other, they play unique differential roles *in vivo*.

**Figure 2:**
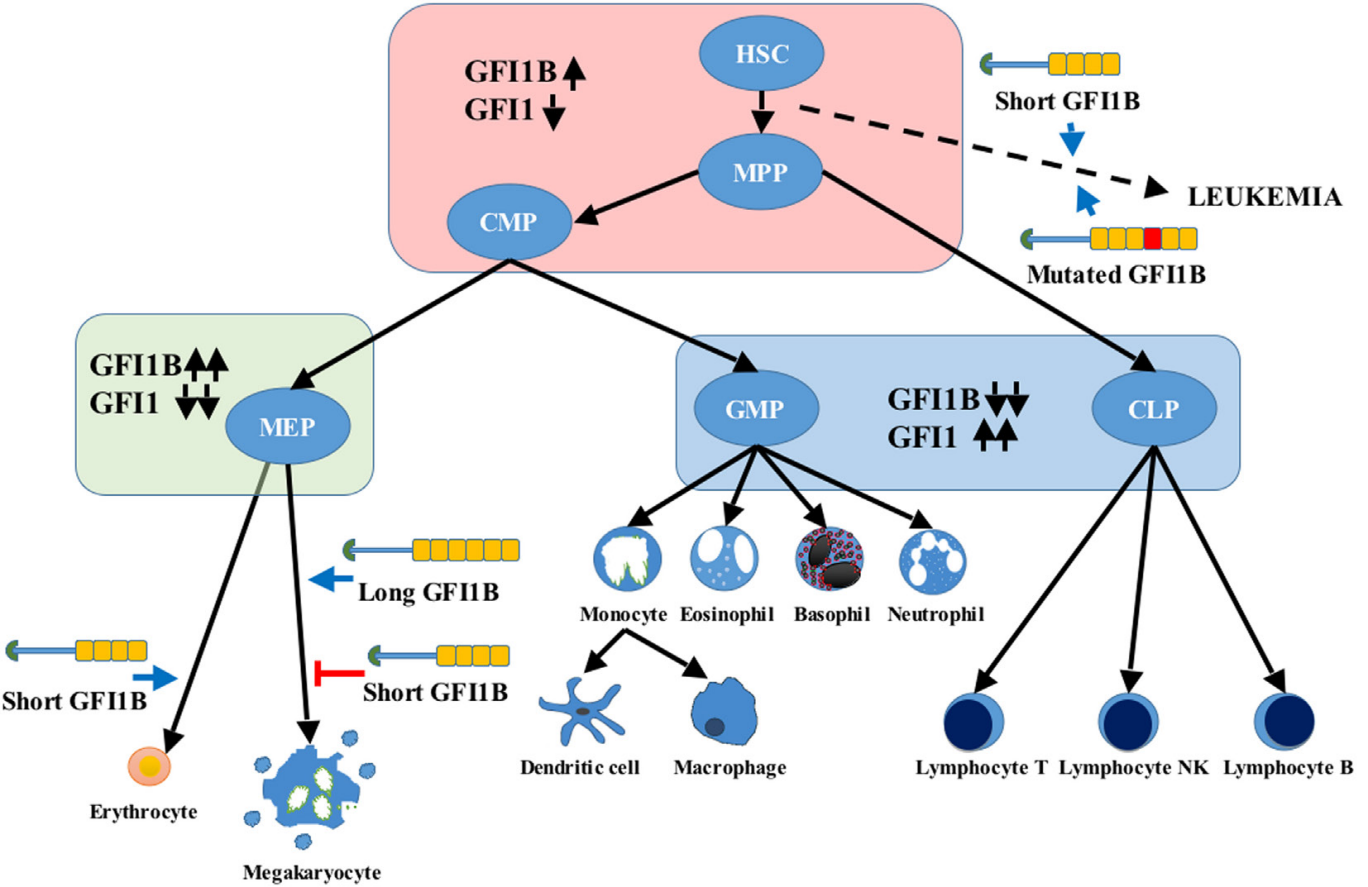
**Differential expression of growth factor independence 1 (*GFI1*) and *GFI1B* during hematopoiesis**. Upward pointing black arrows indicate high expression, and downward pointing black arrows represent low expression. The long and short GFI1B isoforms are represented as in Figure 1. The blue arrows indicate a positive impact on cell differentiation, and the red line reflects an inhibitory effect. Several evidences point to the role of *GFI1B* mutations and increase expression of the short variant in leukemogenesis (dashed arrow). HSC, hematopoietic stem cell; MPP, multipotent progenitor; CMP, common myeloid progenitor; MEP, megakaryocyte and erythroid progenitor; GMP, granulocyte and monocyte progenitor; CLP, common lymphoid progenitor.

Knockout (KO) mice have shown that *Gfi1* is essential for neutrophil differentiation (13, 14); consistently, in humans, severe congenital neutropenia is associated with *GFI1* mutations (15). Gfi1 is also required for B and T lymphopoiesis (Figure 2). Besides being expressed in the hematopoietic system, *Gfi1* is also expressed in precursors of sensory neurons, the retina, specific lung cells, and in the central nervous system (16).

*GFI1B* is expressed in HSCs, common myeloid and megakaryocyte/erythroid progenitors, and erythroid and megakaryocytic lineages. Moderate levels of expression are also found in immature B-cells, a subset of early T-cell precursors (17, 18) and peripheral blood granulocytes and monocytes. GFI1B is very low or absent in lymphoid-primed multipotent, common lymphoid, early thymocyte, and granulocyte–monocyte progenitors (19) (Figure 2).

*GFI1B* role in erythropoiesis is crucial for expansion and differentiation of erythroid progenitors. *Gfi1b* deficiency in mice results in embryonic lethality by day E15 (20). *Gfi1b* null embryos display a delay maturation of erythrocytes and die because of the lack of enucleated erythrocytes. *Gfi1b* KO mice also fail to develop megakaryocytes but have arrested erythroid and megakaryocytic precursors in the fetal liver. Loss of *Gfi1b* in adult mice increases the absolute numbers of HSCs that are less quiescent than wild-type ones (21), ablates erythroid development at an early progenitor stage, and blocks terminal megakaryocytic differentiation in the polyploid promegakaryocytes that fail to produce platelets (22). Lineage-specific KOs have shown that the role of Gfi1b in megakaryocyte polyploidization and motility can be achieved by inhibiting p21 activated kinases, and the effect on proplatelet formation, by controlling α-tubulin expression, which is highly decreased in the KO cells (23).

The short GFI1B variant seems important for erythroid development and to show a stronger repressor activity than the long one (24). Instead, hyperexpression and knockdown experiments in human primary cells have shown that the long GFI1B is required for megakaryopoiesis, but not the short form (25), which may have an inhibitory effect on platelet production (26) (Figure 2).

It has also been observed that the absence of *Gfi1* and *Gfi1b* expression produces a severe block in B-cell development. On the contrary, *in vitro* overexpression of *Gfi1b* inhibits myeloid differentiation of a cultured myelomonocytic cell line (4).

However, GFI1 and GFI1B are not just different in terms of cell specificity, as demonstrated by sequence interchange (27). Consistent with this, *Gfi1* hyperexpression can rescue erythroid and early megakaryocytic differentiation from adult mouse *Gfi1b* KO, but terminal megakaryocyte maturation defect cannot be compensated by *Gfi1* or *Gfi1b* hybrid containing the *Gfi1* N-terminal portion (22). These differences are more patent in the inner ear than in hematopoietic cells (27).

## GFI1B Networks

At the DNA level, GFI1B represses the following: *CDKN1A* (4); proto-oncogenes *myc, myb* (28); *GFI1* (29) and *Meis1* (7); tumor suppressor genes *Socs1* and *Socs2* (30); the antiapoptotic gene *BCL2L1* (*BCXL*) (31); *TGF*β*R3* (32); *GFI1B* itself (29); T lymphopoiesis regulator *GATA3* (33); hematopoietic master regulators *SPI1* (*PU.1*) (34); and *GATA2* (19), among other genes such as *MGEA5, CSDE1, GABPB2*, and *ITSN1* (24). Recent insights into the role of Gfi1b in megakaryopoiesis have revealed new targets repressed by this TF. These include the regulator of G-protein signaling 18, which is differentially regulated in erythroid and megakaryocytic cells, with low expression in the former and high expression in the latter (35). It also represses *Fermt3* (*Kindlin3*) required for integrin-mediated platelet adhesion and *Talin1*, involved in integrin–cytoskeleton connections (36). This is consistent with observations in the megakaryocyte-specific KO model, indicating the importance of Gfi1b control of cytoskeletal and integrin-binding proteins (23).

Besides its repressive function, GFI1B may directly or indirectly activate gene expression; for example, *MEF2C* upregulation in T-cells by GFI1B binding to this gene promoter and *MLLT3*, whose expression correlates with *GFI1B* hyperexpression or functional block (34).

At the protein level, GFI1B interacts with hematopoietic TFs such as SCL, E2A, and GATA1 and with corepressors RUNX1T1 (ETO), CBFA2T3 (ETO2) (37–39), SUV39H1 (40), KDM1A, RCOR1/2, HDACs, and EHMT2.

These findings suggest that *GFI1B* plays a major role in hematopoiesis. Its importance is also reflected by the tight control of its expression. We have identified several evolutionary conserved non-coding elements (CNEs) containing multiple erythroid/megakaryocytic-specific TF-binding sequences, three downstream and one in the first intron (37). Chromatin immunoprecipitation studies in human and mouse demonstrated that GATA1, TAL1 (SCL), NFE2, LDB1, LMO2, SPI1 (PU.1), MYB (37), TCF3 (E2A) (33, 37), and GATA2 (19, 41) are bound to the promoter and/or these distant regulatory elements *in vivo* in human and mouse. This is particularly significant at CNEs +1 and +3, corresponding to mouse DNase1 hypersensitivity sites +13 and +17 (coordinates in relation to the ATG start codon in Kb) (37). These downstream elements have been validated as hematopoietic enhancers in transgenic mouse assays (19). Interestingly, these sites also bind repressors and corepressors GFI1B, CBFA2T3 (ETO2), RCOR1 (COREST), and KDM1A and could behave as negative regulatory elements depending on the GFI1B levels (37).

## Platelet Deficiencies and Bleeding Disorders

Consistent with the *Gfi1b* KO effect on megakaryopoiesis (20, 22, 23), different mutations in *GFI1B* are involved in platelet-related bleeding disorders (42).

A mutation described by Stevenson et al. (43), which consists of a single-nucleotide insertion in *GFI1B* exon 7 (c.880_881insC) that produces a frameshift and protein premature termination (His294fsTer307, g.135866324dupC in GRCh37/hg19 assembly) (Figure 1B: 7), disrupts the integrity of the fifth zinc finger and eliminates the coding sequence for the sixth zinc-finger domain. The mutated protein cannot bind to DNA and loses its repressor activity on target genes. This mutation was described in patients with an inherited dominant bleeding disorder with moderate macrothrombocytopenia and anisopoikilocytosis. Platelets of affected patients had substantial reductions in the α-granule components, P-selectin, and Fg, and somewhat less glycoproteins GPIba and GPIIIa.

Next, Monteferrario et al. (44) detected a nonsense hereditary mutation (c.859C>T) in the amino acid 287 (Gln287Ter, g.135866303C>T in GRCh37/hg19) (Figure 1B: 6) in a family with similar clinical features. This mutation also produces a stop codon and a truncated protein that lacks 44 amino acids at the carboxyl terminus. Normal levels of mRNA are expressed in the mutant allele, but the truncated protein is inactive as a repressor.

The condition produced by both mutations was considered a type of gray platelet syndrome, as platelets look gray under optical microscopy owing to their lack of alpha granules. However, the variable and, in general, less severe α-granule deficiency and the red cell phenotype differ from the classic gray platelet syndrome (45). This has led to classify these conditions as bleeding disorder platelet-type 17 (OMIM #187900).

Recently, another *GFI1B* mutation Gly272fsTer274 (c.814+ 1G>C, g.135865294G>C, GRCh37/hg19) (Figure 1B: 5), generating a truncated protein, has been associated with congenital macrothrombocytopenia linked to α-granule deficiency. This mutation affects 58 amino acids of the C-terminal, which results in complete deletion of zinc finger 5. Platelets in these patients present an increased level of CD34 expression and decreased levels of thrombospondin-1 (46).

*GFI1B* mutations have also been found in two patients from unrelated families with a combined alpha–delta storage pool deficiency, with reduction of α and dense (δ) granules. Both cases had thrombocytopenia. One of them had also anemia and a granulocytic left shift that corrected itself spontaneously in a few months. One patient also had urogenital and heart abnormalities and developed seizures. The other patient had persistent ductus arteriosus. A whole-exon sequencing of the first patient demonstrated a *de novo* heterozygous *GFI1B* nonsense mutation—Lys265Ter (c.793A>T, g. 135865273A>T in GRCh37/hg19) (Figure 1B: 4). Targeted *GFI1B* sequencing in the second case revealed a homozygous mutation at this gene—Leu308Pro (c.923T>C, rs775963992, g.135866367T>C in GRCh37/hg19) (Figure 1B: 8). These mutations too were located at zinc-finger domains 4 and 6, respectively. It is still unclear if the non-hematological congenital abnormalities observed in these patients were related to the *GFI1B* mutations (47).

A GFI1B sequence study of 529 patients with atypical platelet phenotypes also allowed for the identification of seven cases with non-synonymous single-nucleotide polymorphism affecting this locus, which was absent in 11,216 unaffected individuals. Four of them were located at zinc fingers 1 and 2, highlighting the importance of these domains. One of these variants was a homozygous Cys168Phe (c.503G>T, rs527297896, g.135863848G>T in GRCh37/hg19) (Figure 1B: 1) that was associated with abnormal function and reduced platelets in an individual of Asian Indian ancestry; however, the variant was not found in 321 Indian Asian genomes (25).

Whole-exome sequence in 15,459 unselected individuals revealed a synonymous *GFI1B* variant—Phe192 (c.576C>T, rs150813342, g.135864513C>T in GRCh37/hg19, MAF = 0.009) (Figure 1B: 2)—that was associated with low platelet count. Heterozygous carriers had an average platelet reduction of 25–30 × 10^9^/L. This change promotes the short splicing form, affecting megakaryocyte differentiation and platelets, but not red cell production (26).

All these mutations demonstrate the fundamental role of *GFI1B* in the biogenesis of human platelets.

## GFI1B and Malignancy

Mutations that block differentiation and those that promote cell survival or proliferation have been considered necessary for developing acute leukemia (48). Therefore, the major role of GFI1B in hematopoiesis makes it a good candidate to be involved in blood cancers (49). Besides its role in cell differentiation, GFI1B has been reported to possess proapoptotic activity when expressed in human CD34+ cells (50); disruption of this function may also contribute to leukemogenesis.

In keeping with this, *GFI1B* expression has been found in high levels in some primary CD34+ human acute myeloid leukemias (AMLs) and leukemic cell lines. GFI1B silencing in these cell lines decreased proliferation and increased apoptosis (51). In chronic myeloid leukemia (CML), other myeloproliferative neoplasms (MPNs), AML, and B-lymphoblastic leukemias, *GFI1B* expression has also been observed to increase. Remarkably, the short GFI1B isoform is highly expressed in the leukemic cells. However, both isoforms were higher in CML after treating with tyrosine kinase inhibitors (52). Simultaneous silencing of *BCR-ABL1* and *GFI1B* in CML cells showed a cooperative antiproliferative and proapoptotic effect in the K562 CML cell line (53). In this context, the short form may be acting as a repressor over the long species. The caveat of these experiments is the low number of patients and controls analyzed.

*JAK2* V617F mutation is frequent in MPNs but has also been found in the general population (0.14–0.2%). Consistent with the importance of the *GFI1B* downstream sequence in its regulation and the role of this gene in blood cancer, a genome-wide association study identified a C>G variation in this region (rs621940, g.135870130C>G in GRCh37/hg19), which is associated with MPN patients and normal carriers of *JAK2* V617F, but not with normal unmutated individuals (*p* = 1.9 × 10^−7^) (54). Similarly, we reported *GFI1B* promoter mutations in human leukemias. However, no clear link has yet been established between these mutations and hematopoietic neoplasms (55).

Gfi1b repression of oncogene *Meis1* also suggests that GFI1B is involved in leukemia when its repressor function is abolished (7).

In light of these evidences, we described a dominant-negative *GFI1B* mutation, Asp262Asn (c.784G>A, g. 135865264G>A in GRCh37/hg19) (Figure 1B: 3), associated with transition to AML from antecedent myelodysplastic syndrome (MDS). This mutation promotes the survival of normal and MDS human bone marrow CD34+ cells and skews lineage output of these normal adult primary cells and human cord blood common myeloid progenitors toward myeloid cells. This mutant works mainly through master hematopoietic regulator *SPI1* (*PU.1*) (34). In agreement with this, *SPI1* is upregulated in *JAK2* V617F-positive MPNs (56).

Similar to GFI1 (57, 58), GFI1B has been linked to lymphomagenesis. TF TCF3 (E2A) is involved in T-cell human leukemias, and *Tcf3* KO develops T-cell lymphoma. Ectopic expression of *Tcf3* in this context induces growth arrest and apoptosis, together with direct *Gfi1b* upregulation. *Gfi1b*-increased expression in *Tcf3−/−* cells produces similar consequences. Therefore, consistent with the importance of *GFI1B* block in myeloid leukemias, *TCF3* inhibition in T-cell malignancies may work through *GFI1B* downregulation (33). Another piece of evidence of the implication of GFI1B reduction in lymphoma comes from its relation with B-cell lymphoma 6 (*BCL6*), a gene frequently expressed in T- and B-cell lymphomas. *BCL6* chromosomal rearrangements and/or mutations are associated with human lymphomas, up to 73% in diffuse large B-cell lymphoma (59). *Gfi1b* has been identified as a retrovirus integration site in diffuse large B-cell lymphomas of mice containing the human *BCL6* transgene, but this was not the case in retroviral injected non-transgenic control lymphomas. Again, in this context, *Gfi1b* expression was decreased in the first lymphomas compared with the latest. Additionally, GFI1B was decreased in human BCL6-positive T- and B-cell lymphomas, analyzed by immunohistochemistry (60).

Unlike blood malignancies, *GFI1B* and *GFI1* activation has been associated with solid tumors, in particular medulloblastoma. In most cases, *GFI1B*/*GFI1* mutually exclusive abnormal expression was produced by structural variants, showing that abnormal expression of GFI1B is definitely linked with human cancer (61–63). Further investigation may establish a wider role in blood or solid malignancy.

Other genes have been related to malignancy both when upregulated and functionally inactivated, including key regulators of hematopoiesis *CEBPA* (64) and *SPI1* (*PU.1*) (56, 65, 66). This may be the case with *GFI1B* too. However, more data will be needed to get a full insight into the mechanisms involved in *GFI1B*’s role in cancer, particularly in the blood setting and the importance of its two isoforms.

## Author Contributions

All authors listed have made substantial, direct, and intellectual contribution to the work and approved it for publication.

## Conflict of Interest Statement

The authors declare that the research was conducted in the absence of any commercial or financial relationships that could be construed as a potential conflict of interest.
